# Low eating self-efficacy is associated with unfavorable eating behavior tendencies among individuals with overweight and obesity

**DOI:** 10.1038/s41598-023-34513-0

**Published:** 2023-05-12

**Authors:** Noora Oikarinen, Terhi Jokelainen, Laura Heikkilä, Marjukka Nurkkala, Janne Hukkanen, Tuire Salonurmi, Markku J. Savolainen, Anna-Maria Teeriniemi

**Affiliations:** 1 Research Unit of Biomedicine and Internal Medicine, University of Oulu, Oulu, Finland; 2 Department of Medicine, Oulu University Hospital, Oulu, Finland; 3 Medical Research Center Oulu, Oulu University Hospital and University of Oulu, Oulu, Finland; 4 Department of Sports and Exercise Medicine, Oulu Deaconess Institute Foundation sr, Oulu, Finland; 5 Research Unit of Population Health, Faculty of Medicine, University of Oulu, Oulu, Finland; 6 Biocenter Oulu, Oulu, Finland; 7 Research Unit of Health Sciences and Technology (HST), Faculty of Medicine, University of Oulu, Oulu, Finland; 8 Department of Medicine, Endocrinology and Clinical Nutrition, Kuopio University Hospital, Kuopio, Finland

**Keywords:** Health care, Health services, Nutrition, Public health, Weight management

## Abstract

Success in long-term weight management depends partly on psychological and behavioral aspects. Understanding the links between psychological factors and eating behavior tendencies is needed to develop more effective weight management methods. This population-based cross-sectional study examined whether eating self-efficacy (ESE) is associated with cognitive restraint (CR), uncontrolled eating (UE), emotional eating (EE), and binge eating (BE). The hypothesis was that individuals with low ESE have more unfavorable eating behavior tendencies than individuals with high ESE. Participants were classified as low ESE and high ESE by the Weight-Related Self-Efficacy questionnaire (WEL) median cut-off point. Eating behavior tendencies were assessed with Three Factor Eating Questionnaire R-18 and Binge Eating Scale, and additionally, by the number of difficulties in weight management. The difficulties were low CR, high UE, high EE, and moderate or severe BE. Five hundred and thirty-two volunteers with overweight and obesity were included in the study. Participants with low ESE had lower CR (p < 0.03) and higher UE, EE, and BE (p < 0.001) than participants with high ESE. Thirty-nine percent of men with low ESE had at least two difficulties in successful weight control while this percentage was only 8% in men with high ESE. In women, the corresponding figures were 56% and 10%. The risk of low ESE was increased by high UE [OR 5.37 (95% CI 1.99–14.51)], high EE [OR 6.05 (95% CI 2.07–17.66)], or moderate or severe BE [OR 12.31 (95% CI 1.52–99.84)] in men, and by low CR [OR 5.19 (95% CI 2.22–12.18)], high UE [OR 7.20 (95% CI 2.41–19.22)], or high EE [OR 23.66 (95% CI 4.79–116.77)] in women. Low ESE was associated with unfavorable eating behavior tendencies and multiple concomitant difficulties in successful weight loss promotion. These eating behavior tendencies should be considered when counseling patients with overweight and obesity.

## Introduction

Obesity is a growing global chronic public health problem. It is a complex disease with multifactorial etiology: genetic component interacts with psychological, behavioral, and obesogenic environmental factors^1^. Therefore, weight loss interventions need a multidisciplinary approach with psychosocial strategies^2^. Many individuals with overweight and obesity do not receive the counseling they require^3^. Despite a high number of often successful weight-loss attempts, long-term weight loss maintenance remains a remarkable challenge^4,5^. Energy restriction and self-control methods can lead to clinically significant weight loss results in the short run, but these tools can rarely help an individual to achieve sustained weight loss^6^. It has been suggested that the success in long-term weight management depends primarily on psychological and behavioral aspects^7,8^. To develop effective and individualized methods for sustained weight management, we need to further understand the link between psychological factors and eating behavior tendencies.

Self-efficacy is a meaningful and adjustable belief in one’s own capabilities to succeed in certain circumstances despite potential barriers^9^. The concept, originating from Bandura’s social-cognitive theory of human behavior, is widely studied in the field of health behavior. The importance of self-efficacy in predicting health behavior is well recognized^10^. This belief is constructed by previous successes or failures, and it regulates motivation and behavior^11^. Self-efficacy can be general or task-specific^9^, such as eating self-efficacy (ESE)^12^. ESE is defined as confidence to resist eating in challenging circumstances, such as in the presence of negative emotions and in situations with increased food availability and social pressure^12^. ESE plays a central role in long-term weight maintenance^13,14^, since persons with high ESE expectations are more able to engage in favorable lifestyle modifications and maintain the achieved weight-loss compared with individuals with low ESE expectations^13,15–18^. Despite this central role, existing literature is conflicting as to whether success in weight-loss is dependent on the state of ESE^16,19–21^. It has been suggested that ESE is related to differences in clinical response to obesity treatment^22^.

Eating behavior is a multidimensional concept expressing helpful behavior such as cognitive restraint (CR) and maladaptive behavior such as uncontrolled eating (UE), emotional eating (EE), and binge eating (BE)^23,24^. CR describes intentions to restrict intake^25^ and is a method to lose or control weight^24^. UE describes the inability to control eating, leading to eating influenced by external triggers, whereas EE describes the disposition to eat triggered by negative mood states^26^. High tendencies for UE and EE reflect the susceptibility to eat in response to both external and internal cues^27^. BE is characterized by frequent episodes of eating large amounts of food to the point of discomfort^28^. Prior literature shows that scores of UE, EE, and BE are at lower level among individuals who successfully lose or maintain weight than among individuals who fail to achieve these goals^29^.

In weight loss interventions, the level of ESE has often been studied as a predictor of weight loss success^13,16,20,30–32^. However, studies reporting on the association of the level of ESE and eating behavior tendencies are scarce. We aimed to test the hypothesis that individuals with low level of ESE have more unfavorable eating behavior tendencies compared with individuals with high ESE. In addition to individual examination of eating behavior tendencies, our aim was to show that individuals with low ESE have multiple concomitant eating behavior difficulties such as low CR, high UE, high EE, and moderate or severe tendency for BE.

## Methods

This cross-sectional study used data from volunteers enrolled in the Prevention of Metabolic Syndrome (PrevMetSyn, ClinicalTrials.gov Identifier: NCT01959763), a randomized controlled trial from February 2013 to April 2014. The Ethics Committee of the Northern Ostrobothnia Hospital District has approved the original study plan of PrevMetSyn (approval number 29/2012) and all procedures in this trial were performed in accordance with the Declaration of Helsinki (World Medical Association 2013). This study followed the principles of Good Clinical Practice in the execution of the trial. The subjects received both oral and written information, and written informed consent was obtained. A hyperlink to questionnaires was sent to study persons by e-mail after the first study visit and the data collected were analyzed in coded form, i.e., pseudonymized, and pertinent data protection protocols were followed.

### Study design and participants

The PrevMetSyn trial was a one-year randomized clinical trial on the effect of cognitive behavioral therapy-based group counseling and lifestyle counseling via a web-based health behavior change support system (HBCSS). The details of the PrevMetSyn trial have been published previously^33^. The PrevMetSyn trial included 532 participants living in the Oulu area in Northern Finland. The participants of this study were recruited from the Finnish Population Register Center and an invitation letter was sent to 11,400 residents (evenhandedly for both genders) aged 20–60 years. A total of 1065 volunteered for the study and 580 met the eligibility criteria evaluated by a telephone interview. Inclusion criteria were overweight or obesity (body mass index (BMI) of 27–35 kg/m^2^), presence of at least one component of metabolic syndrome, access to the internet, and the ability to use basic information and communications technology such as email and internet. Participants were excluded if they had abnormal laboratory values (thyroid, kidney, and liver function tests) or clinically significant illness with contraindication for weight loss or physical activity. Additionally, exclusion criteria included health-related restrictions to losing weight (e.g., pregnancy), participation in other concurrent weight loss programs, or use of weight loss medications. Power calculation in the PrevMetSyn study was estimated using change in body weight^33^.

### Measures

#### Eating self-efficacy (Weight Efficacy Lifestyle Questionnaire, WEL)

Self-efficacy in relation to eating was assessed by the Weight Efficacy Lifestyle (WEL) questionnaire. WEL is a self-report measure of 20 items^24,34^. It consists of five subscales each including four items relating to negative emotions (e.g., eating when anxious); food availability (e.g., eating on the weekends); social pressure (e.g., eating when others are pressuring to eat); physical discomfort (e.g., eating when in pain); and positive activities (e.g., eating when watching TV). The participants filled in the questionnaire reporting their confidence and ability to resist eating using a 10-point Likert scale ranging from 0 (not confident) to 9 (very confident). Total scores range from 0 to 180, with higher scores indicating stronger ESE beliefs. WEL has been validated to be a reliable measure in samples of weight-loss intervention participants^34^. Cronbach’s alpha was 0.95 for eating self-efficacy in this study.

The participants were divided into two groups based on their score on the WEL questionnaire. We categorized WEL domains as “low ESE” and “high ESE” by using the median as the cutoff point (scores 0–121 = low ESE, scores 122–180 = high ESE). There is no prior literature suggesting an optimal cutoff point for this questionnaire.

#### Eating behavior tendencies (Three Factor Eating Questionnaire-R18, TFEQ-R18 and Binge Eating Scale, BES)

The TFEQ-R18 is a self-report questionnaire consisting of 18 items to assess eating behavior features^24^. The instrument measures three features: CR, UE, and EE. The participants answered 17 items using a 4-point Likert scale for how often they engage in specific eating behaviors and one item using an 8-point Likert scale. CR (six items) is defined as “control over food intake in order to lose weight or prevent weight gain”, UE (nine items) as “overall loss of control in the regulation of eating”, and EE (three items) as “eating in response to negative emotions”. The points of Likert scale were recoded and the sum of every feature (CR, UE, EE) formed the raw score for all features, which were converted to relative proportion (%) of the highest possible raw scores (100%). The scaled scores of each construct range from 0 to 100%, with higher scores reflecting higher intensity of each eating behavior tendency. The TFEQ-R18 is considered to be a reliable measure to describe eating behavior of individuals with obesity^24^. Cronbach’s alphas were 0.65 for cognitive restraint, 0.84 for uncontrolled eating, 0.86 for emotional eating, and 0.87 for binge eating in this study.

The BES is a self-report questionnaire of 16 items to assess the tendency for binge eating^28^. The participants choose a proper alternative from three or four statements. Statements assessed the presence and severity of behavioral, emotional, and cognitive symptoms of binge eating episodes among individuals with overweight or obesity^28,35^. In this study, we applied the cutoff scores of the Finnish Current Care Guidelines of Obesity, where no binge eating is defined with scores 0–19, moderate severity of binge eating with points 20–29, and high severity of binge eating with points 30–46^36^.

#### Defining eating behavior tendencies as difficulties

In addition to examining the scores of each eating behavior tendency, we wanted to consider the intensity of each tendency (CR, UE, EE, and BE) as a difficulty for successful weight control or weight loss. However, the TFEQ-R18 questionnaire and prior literature do not suggest validated cutoff points. Therefore, we divided each TFEQ-R18 tendency into tertiles (low = the tertile with the lowest scores, intermediate = the tertile with intermediate scores, high = the tertile with the highest scores) based on the baseline questionnaire data and used them as categorical variables as follows: low CR (the tertile with the lowest scores, 0–38.9 points) reflecting low attempts to control eating, whereas high UE (the tertile with the highest scores, 51.9–100 points) reflects the susceptibility for external cues for eating and high EE (the tertile with highest scores, 55.6–100 points) reflects the sensibility for internal cues for eating. Additionally, moderate or high severity of binge eating based on the BES questionnaire (merged as one level of difficulty, 20–46 points) was defined as one difficulty. Each of these variables forms one difficulty; the total range of difficulties can therefore vary from 0 to 4.

#### Anthropometric data

The study personnel at the research unit measured height and weight with calibrated equipment. They measured waist circumference with a measuring tape in the horizontal plane midway between the lowest ribs and the iliac crest in standing position on bare skin. They measured resting blood pressure twice after a few minutes of rest. Study nurses drew blood samples after at least ten hours of fasting, and blood samples were analyzed at the clinical laboratory of the Oulu University Hospital (NordLab).

Metabolic syndrome was diagnosed if any three criteria of the following five components were fulfilled: waist circumference ≥ 102 cm in men and ≥ 88 cm in women; serum triglycerides ≥ 1.7 mmol/L or drug treatment for elevated triglycerides; serum HDL < 1.0 mmol/L in men and < 1.3 mmol/L in women or drug treatment for low HDL; blood pressure ≥ 130/85 mmHg or drug treatment for elevated blood pressure; fasting plasma glucose ≥ 5.6 mmol/L, or drug treatment for elevated blood glucose^37,38^.

#### Missing data

The rates of collected data were as follows: anthropometric data 530 out of 532 (missing data 0.4%), WEL questionnaire 469 out of 532 (missing data 11.8%), BES questionnaire 460 out of 532 (missing data 13.5%), and TFEQ-R18 questionnaire 528 out of 532 (missing data 0.8%). Only participants with complete data were included in the analyses when comparing participants with the level of ESE.

### Statistical analyses

All statistical analyses were performed using SPSS Statistics, Version 27 (IBM, Armonk, NY, USA). Continuous variables were presented as means and standard deviations and were analyzed by independent samples T-Test. Categorical variables were expressed as frequencies and percentages for descriptive purposes and were compared by the Pearson Chi-Square Test. A *p* value < 0.05 was considered significant. Normal distribution was evaluated using skewness and kurtosis.

Multiple linear regression analysis using the enter method was conducted to investigate associations between ESE and eating behavior tendencies. ESE was the dependent variable and CR, UE, EE, and BE were the independent variables. Unstandardized coefficients, their 95% confidence intervals (CI), standard errors, and standardized coefficients were reported.

Binary logistic regression analysis using the enter method was performed with low ESE vs. high ESE as the outcome variable. The independent variables were all tertiles of cognitive restraint (the tertile with the highest scores was used as a reference), all tertiles of uncontrolled eating (the tertile with the lowest scores was used as a reference), all tertiles of emotional eating (the tertile with the lowest scores was used as a reference), and all categories of binge eating (no binge eating was used as a reference). Odds ratios and their 95% CIs were reported. There were no significant two-way interaction terms. Goodness-of-fit and explanatory power was assessed with the Hosmer–Lemeshow test and Nagelkerke *R*^*2*^ coefficient, respectively. The assumptions of the regression models were checked. All analyses were stratified by gender.

## Results

### Sample characteristics

Half of the 532 participants were men (51%) and half were women (49%), and about half of the participants were subjects with overweight (48%) and half were living with obesity (52%) (Table 1). Mean age of the participants was 46.0 (SD 10.0) years with no difference in age between the genders. The prevalence of metabolic syndrome was significantly higher among men compared with women (55% vs. 36%, p < 0.001) as previously reported^39^. Women reported higher scores in CR, UE, and EE (p < 0.007) and were more likely to have moderate or high tendency for BE (p < 0.001) than men.

**Table 1:** Baseline characteristics and descriptive statistics of the PrevMetSyn study participants altogether and by gender.

	All Mean (SD) or n (%)	Male Mean (SD) or n (%)	Female Mean (SD) or n (%)	*p* value
Participants	532 (100%)	271 (51%)	261 (49%)	
Age (years)	46.0 (SD 10.0)	45.4 (SD 9.7)	46.6 (SD 10.2)	0.157^a^
BMI (kg/m^2^)	30.4 (SD 2.1)	30.2 (SD 2.1)	30.7 (SD 2.2)	**0.003**^**a**^
Overweight (BMI 27–29.9 kg/m^2^)	255 (48%)	145 (54%)	110 (42%)	**0.009**^**b**^
Obesity (BMI 30–35 kg/m^2^)	277 (52%)	126 (46%)	151 (58%)	
Metabolic Syndrome (yes)	244 (46%)	149 (55%)	95 (36%)	**< 0.001**^**b**^
WEL questionnaire (points) *n* = *469*	120.4 (SD 33.3)	130.7 (SD 30.0)	110.1 (SD 33.2)	**< 0.001**^**a**^
Low ESE (≤ 121)	232 (50%)	85 (36%)	147 (63%)	**< 0.001**^**b**^
High ESE (> 121)	237 (50%)	151 (64%)	86 (37%)	
TFEQ-R18 questionnaire *n* = *528*				
Cognitive restraint (scores)	46.4 (SD 13.8)	44.9 (SD 13.1)	48.1 (SD 14.3)	**0.007**^**a**^
Uncontrolled eating (scores)	44.1 (SD 17.3)	41.1 (SD 16.3)	47.1 (SD 18.0)	**< 0.001**^**a**^
Emotional eating (scores)	44.7 (SD 27.5)	31.4 (SD 23.8)	58.5 (SD 24.3)	**< 0.001**^**a**^
BES questionnaire (points) *n* = *460*	11.4 (SD 7.3)	8.8 (SD 5.4)	13.9 (SD 8.0)	**< 0.001**^**a**^
No binge eating (0–19)	397 (86%)	217 (94%)	180 (79%)	**< 0.001**^**b**^
Moderate binge eating (20–29)	49 (11%)	14 (6%)	35 (15%)	
Severe binge eating (30–46)	14 (3%)	0 (0%)	14 (6%)	
Eating behavior difficulties				
Low cognitive restraint	206 (39%)	115 (43%)	91 (35%)	0.075^b^
High uncontrolled eating	147 (28%)	58 (22%)	89 (34%)	**0.001**^**b**^
High emotional eating	161 (31%)	36 (13%)	125 (48%)	**< 0.001**^**b**^
Binge eating symptoms	63 (14%)	14 (6%)	49 (21%)	**< 0.001**^**b**^

Significant values are in [bold].

SD = Standard Deviation. BMI = Body Mass Index; WEL = Weight Efficacy Lifestyle Questionnaire; low ESE = low eating self-efficacy; high ESE = high eating self-efficacy; BES = Binge Eating Scale; TFEQ-R18 = Three Factor Eating Questionnaire R-18. *p* value is reported between genders. Low cognitive restraint = 0–38.9 points, high uncontrolled eating = 51.9–100 points, high emotional eating = 55.6–100 points, binge eating symptoms = 20–46 points (moderate or high severity).

^a^Independent Samples T-Test.

^b^Pearson Chi-Square Test.

### Eating behavior features by eating self-efficacy categories

The total ESE score ranged between 5 and 180 (median 121) (Table 2). Low ESE was more common in women (63%) than in men (36%, p < 0.001). The first step in the analysis was to examine the association between low ESE and high ESE and the summary scores of each eating behavior tendency. Participants with low ESE reported lower scores than those with high ESE in cognitive restraint (p < 0.027) and considerably higher scores in both uncontrolled eating and emotional eating (p < 0.001). In both genders, participants with low ESE were more likely to have moderate or high tendency for binge eating compared with participants with high ESE (p < 0.001). In both genders, there was no difference between low ESE and high ESE in age, BMI, and in the prevalence of metabolic syndrome.

**Table 2:** Metabolic factors and eating behavior characteristics in the PrevMetSyn study participants with low eating self-efficacy (low ESE) and high eating self-efficacy (high ESE).

Variable	Men			Women		
	Low ESE (n = 85, 36%)	High ESE (n = 151, 64%)	*p* value	Low ESE (n = 148, 63%)	High ESE (n = 86, 37%)	*p* value
Age (years), mean (SD)	45.4 (10.5)	45.7 (8.8)	0.796^a^	45.8 (10.3)	48.1 (9.7)	0.086^a^
BMI, mean (SD)	30.1 (2.0)	30.2 (2.0)	0.676^a^	30.7 (2.0)	30.5 (2.3)	0.489^a^
Metabolic Syndrome, n (%)	45 (53%)	84 (56%)	0.785^b^	46 (31%)	34 (40%)	0.201^b^
TFEQ-R18 questionnaire, mean (SD)						
Cognitive restraint (points)	42.1 (12.8)	46.1 (13.3)	**0.027**^**a**^	43.9 (12.9)	54.2 (12.7)	**< 0.001**^**a**^
Uncontrolled eating (points)	50.8 (11.4)	36.8 (15.9)	**< 0.001**^**a**^	54.1 (16.4)	36.0 (14.0)	**< 0.001**^**a**^
Emotional eating (points)	44.4 (23.2)	25.3 (20.3)	**< 0.001**^**a**^	64.8 (20.4)	46.8 (25.9)	**< 0.001**^**a**^
BES questionnaire (points), mean (SD)	12.7 (5.5)	6.7 (4.0)	**< 0.001**^**a**^	16.6 (8.2)	9.5 (5.3)	**< 0.001**^**a**^
No binge eating	69 (84%)	146 (99%)	**< 0.001**^**b**^	98 (69%)	80 (95%)	**< 0.001**^**b**^
Moderate severity of binge eating	13 (16%)	1 (1%)		31 (22%)	4 (5%)	
High severity of binge eating	0 (0%)	0 (0%)		14 (10%)	0 (0%)	
Eating behavior difficulties						
Low cognitive restraint	43 (51%)	59 (39%)	0.101^b^	68 (46%)	16 (19%)	**< 0.001**^**b**^
High uncontrolled eating	31 (37%)	23 (15%)	**< 0.001**^**b**^	73 (50%)	8 (9%)	**< 0.001**^**b**^
High emotional eating	22 (26%)	8 (5%)	**< 0.001**^**b**^	87 (59%)	26 (31%)	**< 0.001**^**b**^
Moderate or high severity of binge eating	13 (16%)	1 (1%)	**< 0.001**^**b**^	45 (32%)	4 (5%)	**< 0.001**^**b**^

Low ESE = low eating self-efficacy; high ESE = high eating self-efficacy; SD = Standard Deviation; WEL = Weight Efficacy Lifestyle Questionnaire; TFEQ-R18 = Three Factor Eating Questionnaire R-18; BES = Binge Eating Scale; BMI = Body Mass Index. Low cognitive restraint = 0–38.9 points, high uncontrolled eating = 51.9–100 points, high emotional eating = 55.6–100 points, binge eating symptoms = 20–46 points (moderate or high severity).

^a^Independent Samples T-Test.

^b^Pearson Chi-Square Test.

Bold values indicate significance at *P* < 0.05.

### The number of difficulties by ESE categories and gender

Eating behavior tendencies were defined as difficulties in successful weight control or weight loss promotion as follows: low scores of CR (the tertile with the lowest scores), high scores of UE (the tertile with the highest scores) and high scores in EE (the tertile with the highest scores) and moderate or high severity of BE (merged). In men, the most frequent difficulty was low CR in both ESE categories but there was no significant difference between categories (Table 2). In men with low ESE, high UE was more than two times more common, high EE five times more common, and symptoms of BE 16 times more common than in men with high ESE (p < 0.001). In women, the most common difficulty in both ESE categories was high EE (p < 0.001). In women with low ESE, low CR and high EE were twice as common, high UE five times more common, and symptoms of BE six times more common than in women with high ESE (p < 0.001).

**Figure 1:**
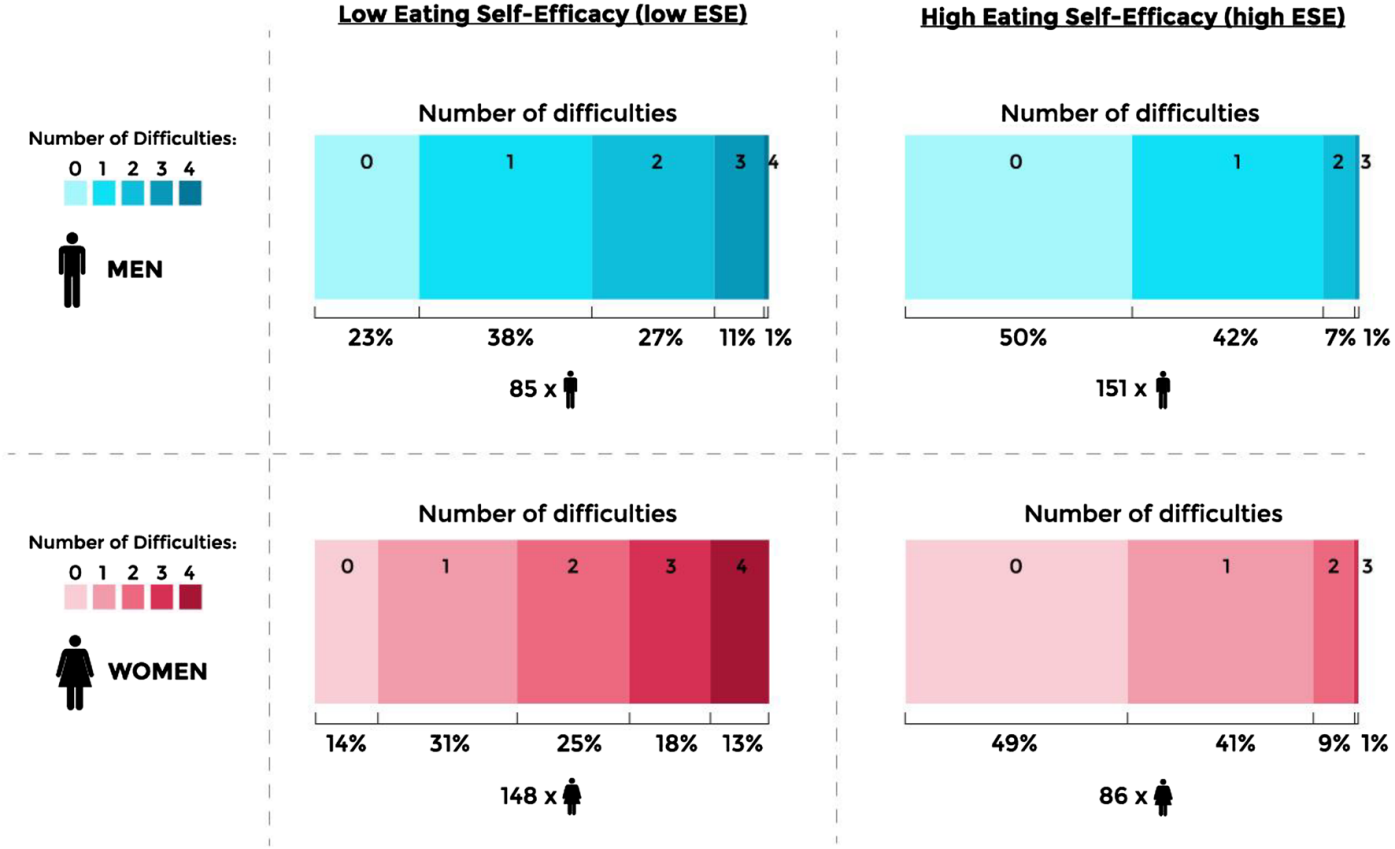
The number of difficulties by eating self-efficacy categories and gender. The difficulties are classified as follows: low cognitive restraint (the tertile with the lowest scores), high uncontrolled eating (the tertile with the highest scores), high emotional eating (the tertile with the highest scores), and moderate or high severity of binge eating (merged). Each of these variables forms one difficulty, therefore the total range of difficulties can vary from 0 to 4. The difference between low ESE and high ESE was tested with Pearson Chi-Square Test, *P* < 0.001.

The next step in data analysis was to examine the concomitant number of difficulties in both low ESE and high ESE stratified by gender. Persons with low ESE in both genders were more likely to have a higher number of eating behavior difficulties compared to subjects with high ESE (p < 0.001, Fig. 1). In men with low ESE, 39% had at least two difficulties, and 12% had three or four difficulties. Furthermore, in women with low ESE, 56% had at least two difficulties, and 31% had three or four. In both genders, half of the subjects with high ESE had no difficulties.

### Eating behavior tendencies associated with ESE

In linear regression analysis, UE, EE, and BE had independent negative associations with ESE (Table 3). Additionally, CR was positively associated with ESE among women (p < 0.001) but not in men (p = 0.711).

**Table 3:** The relationship between eating self-efficacy and eating behavior tendencies in the PrevMetSyn study subjects, i.e., cognitive restraint, uncontrolled eating, emotional eating, and binge eating. Unstandardized coefficients (*B*), standard errors (SE), standardized coefficients (β), 95% confidence intervals (CI), and p values obtained by linear regression analysis.

Variable	Men					Women				
	*B*	SE	(95% CI)	β	*p* value	*B*	SE	(95% CI)	β	*p* value
Cognitive restraint (CR)	0.04	0.11	(− 0.18, 0.26)	0.02	0.711	0.44	0.13	(0.19, 0.69)	0.18	**< 0.001**
Uncontrolled eating (UE)	− 0.32	0.12	(− 0.55, − 0.09)	− 0.17	**0.007**	− 0.63	0.13	(− 0.88, − 0.38)	− 0.34	**< 0.001**
Emotional eating (EE)	− 0.30	0.07	(− 0.44, − 0.16)	− 0.23	**< 0.001**	− 0.23	0.08	(− 0.38, − 0.08)	− 0.17	**0.003**
Binge eating (BE)	− 2.57	0.33	(− 3.23, − 1.91)	− 0.46	**< 0.001**	− 1.03	0.26	(− 1.54, − 0.53)	− 0.25	**< 0.001**

Significant values are in [bold].

The last step in data analysis was to examine the associations of each difficulty and low ESE. In women, intermediate or low scores in CR were associated with a 3- to 5-fold risk of low ESE (p < 0.013, Table 4). In men, intermediate scores in UE were associated with about 5-fold risk of low ESE, and in both men and women, high scores in UE were associated with a 5- to 7-fold risk of low ESE (p < 0.001). In turn, in women, intermediate scores in EE were associated with a 12-fold risk of low ESE, and in both men and women, high scores in EE were associated with a 6- to 24-fold risk of low ESE (p < 0.002). Given the small size of the group with high severity of BE, moderate and high severity of BE were merged as one category. In men, moderate or severe BE was associated with a 12-fold risk of low ESE (p = 0.019).

**Table 4:** The relationship between low eating self-efficacy and eating behavior tendencies, i.e., cognitive restraint, uncontrolled eating, emotional eating, and binge eating in the PrevMetSyn study. Unstandardized coefficients (*B*), standard errors (SE), odds ratios (OR), 95% confidence intervals (CI), and p values obtained by binary logistic regression analysis using the enter method.

Variable (points)	Men				Women			
	*B*	SE	OR (95% CI)	*p* value	*B*	SE	OR (95% CI)	*p* value
CR high (50–100)			Reference				Reference	
CR intermediate (39–49.9)	0.15	0.45	1.16 (0.49–2.79)	0.733	1.07	0.43	2.92 (1.25–6.81)	**0.013**
CR low (0–38.9)	0.49	0.42	1.63 (0.72–3.68)	0.244	1.65	0.44	5.19 (2.22–12.18)	**< 0.001**
UE low (0–37)			Reference				Reference	
UE intermediate (37.1–51.8)	1.57	0.45	4.83 (1.87–11.65)	**0.001**	0.63	0.41	1.87 (0.83–4.20)	0.129
UE high (51.9–100)	1.63	0.51	5.37 (1.99–14.51)	**0.001**	1.97	0.53	7.20 (2.41–19.22)	**< 0.001**
EE low (0–33.3)			Reference				Reference	
EE intermediate (33.4–55.5)	0.70	0.37	2.00 (0.97–4.16)	0.060	2.50	0.79	12.23 (2.59–57.81)	**0.002**
EE high (55.6–100)	1.80	0.55	6.05 (2.07–17.66)	**0.001**	3.16	0.82	23.66 (4.79–116.77)	**< 0.001**
No BE (0–19)			Reference				Reference	
Moderate or severe BE (20–46)	2.51	1.07	12.31 (1.52–99.84)	**0.019**	1.03	0.63	2.80 (0.82–9.55)	0.100

Significant values are in [bold].

CR = cognitive restraint; UE = uncontrolled eating; EE = emotional eating; BE = binge eating; low = the tertile with lowest scores, intermediate = the tertile with intermediate scores; high = the tertile with highest scores.

Linear regression models were significant among men (R^2^ = 0.49, F(4, 224) = 54.56, p < 0.001) and women (R^2^ = 0.50, F(4, 222) = 54.75, p < 0.001). In men, the logistic regression model using the enter method including eating behavior tendencies, i.e., cognitive restraint, uncontrolled eating, emotional eating, and binge eating as variables to be associated with low ESE was statistically significant, χ^2^ (7, N = 229) = 67.14, p < 0.001. The model explained 34.9% (Nagelkerke R square) of the variance in the dependent variable and correctly classified 76.4% of the cases. In women, the entered logistic regression to analyze low ESE was statistically significant χ^2^ (8, N = 227) = 94.59, p < 0.001. The model explained 46.5% (Nagelkerke R square) of the variance in the dependent variable and correctly classified 59.0% of the cases.

## Discussion

Our main result was that individuals with low ESE had more often unfavorable eating behavior tendencies and more concomitant eating behavior difficulties than individuals with high ESE. This finding might help to understand the psychological and behavioral aspects behind obesity. Those who reported low intensity of cognitive restraint (CR) or high intensity of uncontrolled eating (UE), emotional eating (EE), or binge eating (BE) had higher risk of experiencing low confidence to resist eating in challenging circumstances.

Previous studies show that low ESE is associated with UE and EE^40,41^ and binge eating symptoms^22,40,42^. Our results are in line with these findings. However, in previous studies eating behavior tendencies have been investigated separately in relation to ESE^30,40,41^ but their concomitant association with ESE was not studied. Therefore, we examined the co-existence of multiple eating behavior difficulties and their association with ESE. Our finding that individuals with low ESE in both genders were more likely to have a higher number of eating behavior difficulties compared to subjects with high ESE is novel. Previously, individuals with low scores in CR and high scores in UE and EE were described as a group with low efforts to control eating and who are susceptible to external and internal triggers to eating^27^. Additionally, high scores in CR combined with high scores in UE and EE was recognized as a detrimental combination because of its association with poorer food choices and coping strategies. These findings suggest the relevance of the results of our study.

Future studies should investigate which eating behavior clusterings are harmful and which are protective in terms of ESE. Moreover, the associations of eating behavior clustering and weight need future studies because Pentikäinen et al.^27^ did not report the BMI of study subjects, whereas in our study the population was composed of individuals with overweight and obesity. Acknowledging the coincidental eating behavior difficulties and their prevalence as well as the level of ESE may help to improve the fit between the individual and weight management programs^43^.

Gender seems to affect ESE as the prevalence of low ESE was almost 2-fold in women compared to men. In previous studies, however, conflicting results on gender differences in ESE have been reported^30,44,45^. In addition to women having much higher prevalence of low ESE in comparison to men, women also had higher risk of multiple concomitant eating behavior difficulties. These results might be explained by gender differences in eating behavior, which are in line with previous results^46,47^. Most of women with low ESE tend to have difficulties with eating control and are susceptible for challenges in regulation and lack of the ability to resist eating. Most of men with low ESE may turn out to be susceptible for challenges in regulation and lack of the ability to resist eating, however, less frequently than women with low ESE.

As previously reported, ESE has varied in participants in weight-loss interventions^30^, therefore it is possible that in some individuals high ESE and high BMI may also be concurrent. Many participants in weight-loss interventions have usually had previous weight loss attempts, which may explain the wide variety in ESE. Therefore, our finding of association of BMI and ESE may not exclude the possibility that people with high ESE might succeed better in weight loss, as previously reported. The latter may have significance in clinical setting*.*

In clinical practice, besides BMI, metabolic markers, dietary quality, and demographic information, psychosocial variables such as self-efficacy are important in order to form a holistic view of a patient´s situation. Based on the results of this study, it is equally relevant to recognize individuals with low ESE and those with high ESE in order to provide tailored counseling. This underlines the importance of health care professionals’ competence to recognize psychological factors affecting eating as well as valid tools to recognize different tendencies and capabilities of the patient in order to provide tailored care. One possible alternative improving self-efficacy and favorable eating behavioral tendencies might be cognitive behavioral techniques which enhance patients’ competence autonomy and intrinsic motivation^2^.

Our study has some important strengths including a large population-based study sample, a high number of men, and a diverse combination of eating behavior factors. Additionally, the rates of missing data are low. Therefore our results may be generalizable to people seeking treatment for obesity, at least in Western countries. The most notable limitation concerns the use of a dichotomous split for the ESE. This approach might affect statistical power and the categorization might be too strict. However, there is no prior literature suggesting an optimal cutoff point for this questionnaire. Therefore, multiple linear regression analysis using the enter method was also conducted to investigate associations between ESE and eating behavior tendencies. Another limitation is the cross-sectional nature of the analysis, which rules out causal conclusions.

## Conclusions

Low ESE was associated with unfavorable eating behavior tendencies and multiple concomitant eating behavior difficulties to successful weight loss promotion. These eating behavior tendencies associated with low ESE should be considered when counseling patients with overweight and obesity. Additionally, the competence of health professionals to recognize and provide tailored care for individuals with feelings of low capabilities and unfavorable eating behavior tendencies may have an impact on the response to successful weight management. More research is needed on which tailored obesity treatment approaches are the most effective for individuals with low ESE and multiple concomitant eating behavior difficulties.

## Data Availability

The datasets used and/or analyzed during the current study are available from the corresponding author on reasonable request.
